# Supplementary material for: FERN – a Java framework for stochastic simulation and evaluation of reaction networks
Source: BMC Bioinformatics. 2008 Aug 29;9:356. doi: 10.1186/1471-2105-9-356 (PMC2553347; doi:10.1186/1471-2105-9-356)
Supplement: Additional file 1 — FERN distribution, Version 1.3. This archive contains the FERN source code and binaries as well as documentation and example models in FernML and SBML. [file 1471-2105-9-356-S1.zip › fern/doc/javadoc/fern/simulation/class-use/Simulator.FireType.html]

Uses of Class fern.simulation.Simulator.FireType


---


|  |  |  |
| --- | --- | --- |
| **Overview** \| **Package** \| **Class** \| **Use** \| **Tree** \| **Deprecated** \| **Index** \| **Help** | |  |
| PREV   NEXT | **FRAMES**    **NO FRAMES**     **All Classes** |  |


---


## **Uses of Class fern.simulation.Simulator.FireType**

| Packages that use Simulator.FireType | |
| --- | --- |
| **fern.cytoscape** | Provides the classes for the cytoscape plugin. |
| **fern.example** | Contains examples and demonstrations of the framework. |
| **fern.network.sbml** | Provides the classes for parsing and using sbml based networks. |
| **fern.simulation** | Provides algorithms for simulating reaction network as well as methods for observer certain aspects of a simulation. |
| **fern.simulation.observer** | Provides classes to observe certain aspects of simulations. |

Uses of Simulator.FireType in fern.cytoscape

| Methods in fern.cytoscape with parameters of type Simulator.FireType | |
| --- | --- |
| `void` | `CytoscapeColorChangeObserver.activateReaction(int mu, double tau, Simulator.FireType fireType, int times)` |

Uses of Simulator.FireType in fern.example

| Methods in fern.example with parameters of type Simulator.FireType | |
| --- | --- |
| `void` | `CellGrowthObserver.activateReaction(int mu, double tau, Simulator.FireType fireType, int times)` |

Uses of Simulator.FireType in fern.network.sbml

| Methods in fern.network.sbml with parameters of type Simulator.FireType | |
| --- | --- |
| `void` | `SBMLEventHandlerObserver.activateReaction(int mu, double tau, Simulator.FireType fireType, int times)` |

Uses of Simulator.FireType in fern.simulation

| Methods in fern.simulation that return Simulator.FireType | |
| --- | --- |
| `static Simulator.FireType` | `Simulator.FireType.valueOf(String name)`             Returns the enum constant of this type with the specified name. |
| `static Simulator.FireType[]` | `Simulator.FireType.values()`             Returns an array containing the constants of this enum type, in the order they are declared. |

| Methods in fern.simulation with parameters of type Simulator.FireType | |
| --- | --- |
| `protected  void` | `Simulator.fireReaction(int mu, double t_start, double t_end, int times, Simulator.FireType fireType)`             Fires a reaction. |
| `protected  void` | `Simulator.fireReaction(int mu, double t, Simulator.FireType fireType)`             Fires a reaction. |

Uses of Simulator.FireType in fern.simulation.observer

| Methods in fern.simulation.observer with parameters of type Simulator.FireType | |
| --- | --- |
| `void` | `ReactionIntervalObserver.activateReaction(int mu, double t, Simulator.FireType fireType, int times)`             Record the firing of mu, if mu is registered. |
| `void` | `RandomNumberGeneratorCallObserver.activateReaction(int mu, double tau, Simulator.FireType fireType, int times)`             Do nothing. |
| `abstract  void` | `Observer.activateReaction(int mu, double tau, Simulator.FireType fireType, int times)`             Gets called before a reaction fires. |
| `void` | `LeapObserver.activateReaction(int mu, double tau, Simulator.FireType fireType, int times)`             Do nothing. |
| `void` | `IntervalObserver.activateReaction(int mu, double tau, Simulator.FireType fireType, int times)`             Do nothing. |
| `void` | `InstantOutputObserver.activateReaction(int mu, double tau, Simulator.FireType fireType, int times)`             Prints event. |
| `void` | `FireTypeObserver.activateReaction(int mu, double tau, Simulator.FireType fireType, int times)`             Counts the firing for the given `Simulator.FireType` |
| `void` | `AmountAtMomentObserver.activateReaction(int mu, double tau, Simulator.FireType fireType, int times)`             Do nothing |

---


|  |  |  |
| --- | --- | --- |
| **Overview** \| **Package** \| **Class** \| **Use** \| **Tree** \| **Deprecated** \| **Index** \| **Help** | |  |
| PREV   NEXT | **FRAMES**    **NO FRAMES**     **All Classes** |  |


---
